# Hepatitis B And C In Hematopoietic Stem Cell Transplant

**DOI:** 10.4084/MJHID.2009.016

**Published:** 2009-12-03

**Authors:** Anna Locasciulli, Barbara Montante, Emanuela Morelli, Virginia Gulino, Anna Proia, Maria Beatrice Pinazzi

**Affiliations:** Pediatric Hematology Hematology Departments, San Camillo Hospital, Circonvallazione Gianicolense 87, 00152 Rome, Italy

## Abstract

Although the risk of acquisition of hepatitis B or hepatitis C virus through blood products has considerably reduced since the last decade, some infected patients are candidates to stem cell transplantation. Others may have no alternative than an infected donor. In all these cases, recipients of transplant are prone to short and long term liver complications. The evolution of liver tests under chemotherapy before transplant may give useful information to anticipate on the risk of hepatitis reactivation after transplant, both for HBv and HCv. More than sixty percent of the patients who are HBsAg-positive before transplant reactivate after transplant, and 3% develop acute severe liver failure. Because both viral replication and immune reconstitution are the key factors for reactivation, it is crucial to closely follow liver function tests and viral load during the first months of transplant, and to pay a special attention in slowly tapering the immunosuppression in these patients. Lamivudine reduces HBv viremia, but favors the emergence of HBv polymerase gene mutants and should be individually discussed. Both in case of HBv or HCv hepatitis reactivation with ALT ≥ 10N concomitantly to an increase in viral load at time of immune reconstitution, steroids should be given. In case there is no alternative than a HBv or HCv positive geno-identical donor, the risk of viral hepatitis, including acute liver failure and late complications, should be balanced with the benefit of transplant in a given situation.

## Introduction:

The occurrence of infection with Hepatitis B (HBV) or Hepatitis C virus (HCV) in patients undergoing allogeneic or autologous stem cell transplantation (HSCT) poses several clinical problems, as these infectious complications can jeopardize the ultimate prognosis, due to the possibility of progression to fulminant hepatic failure and also to possible evolution to chronic active hepatitis and cirrhosis. Although the risk of acquiring HBV and HCV infection from blood transfusion is nowadays greatly reduced, HSCT patients still represent a group at high risk, being prone to becoming infected due to the lack of immune competence given both the hematological disease and the conditioning regimen they receive before HSCT. Moreover, these patients may already be infected at transplant. Lastly, patients undergoing allogeneic HSCT may have a single related donor who is an HBV or HCV carrier, and this event is far from exceptional, especially in geographic areas where these viruses are endemic. The behavior of HBV and HCV infection and related disease is often unpredictable in this cohort of patients both in the short-term and long-term outcome.

## Hepatitis B Virus Infection and Related Liver Disease

### Biology and Pathogenesis of Hepatitis B virus:

HBV is a DNA virus classified in the EPADNA Virus Family. Its molecular organization and replication mechanisms have been extensively characterized 1. The virus replicates in hepatocytes with high efficiency and viral replication produces a large amount of viral particles with high level of viraemia. HBV is also able to integrate its genome into the host DNA and produces a number of structural and non-structural proteins which modulate the virus-cell interactions. Some of these proteins have regulatory and transcriptional functions which control gene expression and may be involved in hepato-carcinogenesis. The replicative phase of HBV infection is characterized by the presence of a soluble viral protein (HbeAg) and of HBV-DNA in serum. Seroconversion to anti-Hbe characterizes the transition from the replicative into the non-replicative phase (integration phase) and is usually associated with disappearance of viraemia. These events have clinical relevance as they associate with remission of liver-cell damage, indicating that virus replication is a pre-requisite for triggering hepato-cellular injury. Complete recovery from HBV infection is associated with seroconversion from HbsAg positive to antiHBs positive status. In the past decade, accumulating evidence indicates that some patients may become chronically infected with HBV with persistent virus replication in the absence of HbeAg in serum. These cases are infected by HBV pre-core mutants that replicate persistently while not secreting HbeAg, due to point mutations in the coding region. HBV is not considered to be directly cytopathic in the immune-competent host and most evidence supports the conclusion that the host-immune response plays a major role in the pathogenesis of HBV-related liver damage. However, in the presence of exceptionally high virus replication and expression of virus products in the infected cells, a direct cytopathic mechanism may also supervene^2^,^3^. The current interpretation of HBV immune-pathology explains liver-cell damage as the result of reactivity of virus-specific cytotoxic T lymphocytes recognizing epitopes of the core or the envelope antigens on the surface of infected hepatocytes. The release of high concentration of soluble cytokines in the liver may also contribute to amplification of liver damage. The pathogenesis may be somehow different in patients infected by HbeAg negative pre-core mutants, which are often associated with more severe flare-ups of liver damage.

### Natural course of hepatitis B in the Immunocompetent Host:

HBV infection is responsible for acute hepatitis, which may develop into a fulminant course in 1% of cases, or progress to chronic infection in 5–10%. Chronic hepatitis B shows variable histological activity, that may eventually lead to cirrhosis in 20–40% of patients. Further progression includes decompensated liver disease and death for liver-related causes. Carriers of HBV have a 300-fold increased risk of developing hepatocellular carcinoma (HCC). Most HBV carriers, however, have never developed severe liver complications during their lifetime. The current treatment for chronic hepatitis B is based on the use of interferon and nucleotide analogues (mostly lamivudine, but also adefovir, lobucavir and others). The sustained response rate after 6 months of treatment is between 20 and 40%. However, eradication of infection is rarely achieved and HBV often reactivates when therapy is withdrawn.

### Acute events and risk factors of HBV infection early post Hematopoetic Stem Cell Transplantation (HSCT):

The prevalence of HBV infection in patients undergoing HSCT differs according to geographic areas, and ranges from 1% to 28% in published reports (Table 1)^5^–^9^. In a prospective study including 15 HSCT European units, six out of 193 patients (3%) treated consecutively were found HbsAg positive before HSCT^10^ thus confirming that HBV infection still represents a clinical problem in this setting.

An acute exacerbation of liver disease following immune reconstitution occurs in more than 60% of cases. Data derived from a multicenter study^11^ show that the risk of hepatitis B reactivation at 24 months after HSCT was 81% for allogeneic and 66% for autologous cases (p = 0.3). As to the timing, the majority of patients had a clinical exacerbation of liver damage within the first year post-transplant. Interestingly, in the autologous group a majority of patients showed ALT peaks within six months after HSCT, while most patients undergoing allogeneic transplant did so later on. Reactivation can be followed by complete recovery and seroreversion to HbsAg negative status in one fourth of cases, even without antiviral therapy^12^, while a fulminant evolution occurs in a smaller proportion of patients (3%) As to the outcome of post-SCT hepatitis, the majority of reports indicates that the disease is often mild and asymptomatic, with moderate but long-lasting transaminase abnormalities^5^,^6^,^7^. The profound impairment of the immune response can also influence the serological profile, which is often bizarre: in HbsAg positive patients, serum antibodies to HbcAg are frequently absent, even in cases with high-level HBV replication and also in some cases receiving a bone marrow from an anti-HBc positive donor^5^. Failure to develop anti-HBc in these cases may be the consequence of intrinsic B-cell defect or of excessive suppressor T cell activity, which are known to deeply influence the process of immune reconstitution following SCT. On the other hand, a transient appearance of HBV antibodies after transplant is frequent, also in cases with no evidence of HBV infection, likely due to passive immunization via blood products administered: in a pediatric prospective study we observed that more than 90% of children had detectable antibodies in serum after transplant, which disappeared during follow-up in more than half of them^13^.

### “De novo” HBV infection: risk factors and related liver disease:

Patients undergoing HSCT can develop a “de novo” HBV infection post-transplant, and the overall frequency of such a complication, diagnosed by means of HbsAg and HBV-DNA seroconversion, was reported to be around 3%^10^. Sometimes the origin of the infection is evident, such as in case of positive stem cell donors, but in the majority of cases the modalities of transmission are less obvious, especially considering that all blood products are nowadays screened for HBV serum markers. In a recent study including 24 patients receiving HBV infected marrow, HbsAg became detectable in 22% of pre-BMT negative recipients, but only 5.5% became chronic carriers^24^. Antigenemia developed more frequently in anti-HBs negative compared with anti-HBs positive patients. Fatal liver failure occurred in 21% of cases. Patients with an anti-Hbe donor had a higher frequency of liver failure (28% versus 0%) and higher transaminase peak values compared to HbeAg positive donors. Liver failure was never observed in anti-HBs positive recipients. The observation that the rate of seroconversion is lower than expected in case of positive donor was confirmed in a prospective study by the Infectious Diseases Working Party of the European Blood and Marrow Transplantation Group^10^. Of four patients receiving BMT from an HbsAg positive donor, only two became HbsAg positive. Liver disease in these patients was minimal and none suffered from fulminant hepatitis. In the same study, the prevalence of “de novo” HBV infection in patients transplanted with an HbsAg negative donor was 2%, and also in these patients, the HBV-related liver disease was clinically benign. The observation that before HSCT 2 out of 13 anti-HBc positive patients became HbsAg positive during follow-up, compared to 2 out 157 anti-HBc negative cases, poses the question whether these episodes were true “de novo” rather than reactivation of latent infection, an event already reported in the literature^25^.

### Long-term sequelae of Hepatitis B infection after Hematopoietic Stem Cell Transplantation:

Patients infected with HBV generally show a mild to moderate liver disease on long-term follow-up, and cirrhosis due to chronic B hepatitis has not been reported to date.

It should be stressed that flares in transaminases after HSCT, even in the presence of viraemia, doesn’t automatically translate in viral hepatitis reactivation, as other causes, such GVHD, iron overload, and other infections, can result in this clinical picture. In these cases, liver biopsy should be considered,

## Hepatitis C Virus Infection And Related Liver Disease

### Biology and pathogenesis of Hepatitis C virus:

HCV is a double-stranded RNA virus classified within the Flaviviridiae. The virus consists of a family of closely related while genetically distinct types and subtypes. Six major genotypes have been identified and classified, from HCV1 to HCV6. The mechanisms of replication of HCV and pathogenesis of the associated liver disease have been only partially characterized. The infection has a high propensity to becoming chronic, due to the high mutation rate of the envelope region coding for the surface antigens allowing the virus to easily escape to the host-immune response. As for HBV, liver damage in hepatitis C is thought to be dependent mainly on the host-immune response mediated by cytotoxic T cells. HCV infection is responsible for hepatic and extra-hepatic disease. The extra-hepatic manifestations include chronic fatigue, cryoglobulinemia, vasculitis and autoimmune disorders. These manifestations are most likely due to direct interaction of HCV with the immune system through the CD81 receptor^4^. Monoclonal expansion of B cells may occur, leading to low-grade B-cell lymphoma. HCV replication is significantly increased by immunosuppression and, as in hepatitis B, it may trigger a direct cytopathic effect in infected cells. Immune complexes-mediated immunopathology may also be relevant in hepatitis C.

### Natural course of hepatitis C in Immunocompetent host:

Acute hepatitis C is often asymptomatic and mild and is rarely recognized clinically. A fulminant or severe course is exceptionally rare in the absence of cofactors or of coinfections. On the other hand, chronic carriers of HCV might be particularly prone to develop fulminant hepatitis when superinfected by Hepatitis A Virus (HAV) or other hepatotropic agents. Hepatitis C becomes chronic in 60–80% of infected individuals and very few of them are healthy chronic carriers, while most show histological evidence of chronic hepatitis. Chronic hepatitis progresses to cirrhosis in 20–40% of the cases within 10–20 years after onset. The major factors that increase the risk of progression are: age, alcohol abuse, coinfection with HBV or HIV, immunosuppression, and iron overload. After cirrhosis has developed, decompensation occurs and HCC may develop with an annual incidence of 3%.

The current treatment for hepatitis C is based on the combination of Interferon alpha with the nucleotide analogue Ribavirin for 12 months. This therapy produces a rate of sustained response ranging from 10% to 40%, depending on the HCV genotype and pretreatment virus load.

**Figure 2.**
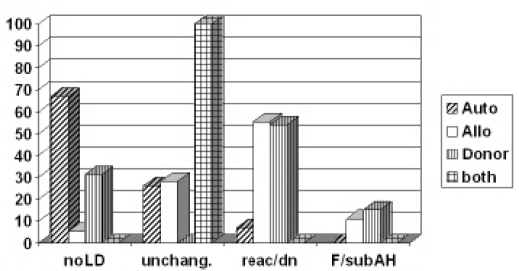
Post-SCT liver disease in HCV infection.

Before the routine screening of blood donors was introduced, the prevalence of HCV infection in HSCT recipients, based on the detection of specific antibodies, was reported to range from 10% up to 30%^13^,^14^,^15^. These figures certainly underestimated the problem, as in these patient populations the diagnostic sensitivity of anti-HCV is particularly low in the presence of immunosuppression, thus the only method to identify HCV infection is the detection of HCV-RNA. Although the risk of transfusion-associated transmission has dramatically decreased in the last decade, the problem is still present. In a pediatric series, 25% of children transplanted before 1990 - when HCV screening for blood donor was introduced in Italy - were found anti-HCV positive, but afterwards 15% of cases were still found infected^16^. More recently, results derived from a prospective study showed that 6% of HSCT candidates were HCV-RNA positive before transplant: only half of them were also positive for anti-HCV^10^. The identification of pre-transplant HCV infection appears clinically relevant: besides the possibility of viral reactivation and hepatitis exacerbation following immune reconstitution and the risk of chronic evolution and cirrhosis, being infected with HCV has been indicated as an independent risk factor for post-transplant veno-occlusive disease of the liver^17^,^18^. This relationship, however, is controversial, as other investigators did not find such an association either in retrospective or in prospective studies including both pediatric and adult populations^10^,^13^,^19^,^20^,^21^. Reactivation of chronic HCV infection after tapering immunosuppressive medication, sometimes leading to fulminant hepatic failure, has also been reported^22^,^23^, thus emphasizing the role of an immune mediated mechanism in the patho-genesis of HCV-related liver disease. Data derived from an Italian multicenter study^11^ show that the risk of hepatitis C reactivation at 24 months after HSCT was 100% for allogeneic and 16% for autologous cases^11^. Figure 2 shows the type of post-HSCT liver disease occurring in HCV infected patients undergoing autologous or allogeneic HSCT. The risk of death after reactivation was 8%, and was observed only in allogeneic setting, while the probability of complete resolution of liver disease after reactivation was 67% and 48% in allo or autologous transplant respectively^11^. When HBV and HCV positive patients were compared, some interesting differences in the ALT profiles following auto-logous and allogeneic HSCT were observed. In HCV positive cases, ALT elevation was more frequent, severe and protracted following allogeneic graft compared to autologous transplant. When the course of reactivation was benign, recovery from liver disease was more frequently seen in allogeneic compared to autologous HSCT. Therefore in HCV infection other factors may influence the profile of liver disease, such as immune suppression, conditioning regimen and GVHD prophylaxis, donor immunity and, possibly, the GVH reaction.

### “De novo” HCV infection, risk factors and related liver disease:

Patients undergoing HSCT can develop a “de novo” HCV infection post-transplant, and the overall frequency of such a complication, diagnosed by means of HCV-RNA seroconversion, was reported to be around 7%^10^. Sometimes the origin of the infection is evident, such as in case of positive stem cell donors, but in the majority of cases the modalities of transmission are less obvious, especially considering that all blood products are nowadays screened for HCV serum markers The prevalence of “de novo” HCV infection in the latter study was around 7%: in 2 cases the infection was transmitted by a positive stem cell donor^10^. Interestingly, only 2 out of 4 patients receiving an infected marrow became viraemic, while Shuhart et al. have observed a 100% rate of transmission in such cases^14^. Shuhart also reported that viraemia occurred within a few days after HSCT, none cleared the virus and the acute illness was subclinical in all cases while in our study we observed that seroconversion occurred within 15–180 days, with 40% of patients suffering from severe hepatitis being fatal in one case^10^. When the transplant procedure can be postponed, HCV transmission via stem cells may be prevented by treating the donor with interferon, that could eliminate viremia within three months^26^. However, the withdra of such a therapy at least two weeks before harvest should be considered, as interferon might influence HSCT outcome, affecting both quality of SC harvest and take^27^,^28^.

These contradicting data indicate that more information is needed to carefully establish firm conclusions and recommendations in this special patient setting, and that the risk in individual patients is difficult to predict.

### Long-term sequelae of Hepatitis C infection after hematopoietic Stem cell Transplantation:

Patients infected with HCV generally show a mild to moderate liver disease on long-term follow-up. Chronic C hepatitis, it is often asymptomatic, with biochemical abnormalities characterized by fluctuation in transaminase levels, ranging from normal to abnormal values over time, with no signs or symptoms of decompensated liver disease at least during the first decade following HSCT^13^,^18^,^19^,^20^. Recently, the Seattle group has reported a progression to cirrhosis and decompensated liver disease in some patients observed for more than ten years 18,27. The Saint Louis group published results on 96 patients infected with HCV between 1973 and 1995, with a median follow-up of more than 15 years: the cumulative incidence of progression to cirrhosis was 11% at 15 years and 24% at 20 years. Genotype 3 was found an additional risk factor, while extrahepatic manifestations, such as cryoglobulinemia, thrombocytopenia, neurological, and renal manifestations, had no significant impact.^28^.

It should be stressed that, also in patients with HCV infection, (like those with HBV) flares in transaminases after HSCT, even in the presence of viraemia, doesn’t automatically translate in viral hepatitis reactivation, as other causes, such GVHD, iron overload, and other infections, can result in this clinical picture. In these cases, liver biopsy should be considered,

These clinical observations indicate that, although the outcome of this chronic hepatitis does not seem to be significantly influenced by transplant procedures, it may represent an important clinical problem in long-term survivors.

-Whenever possible, pretransplant liver biopsy should be considered in case of clinical evidence of chronic liver disease, alcohol abuse (which enhances the risk of hepatic fibrosis), documented long-lasting active C hepatitis (more than 10 years).
-HSCT patients who are HbS Ag positive or HCV positive, or who received a transplant from a positive HBV or HCV donor should be closely monitored for liver function tests (LFT’s), and especially transaminases, and HBV or HCV serum markers at least every two weeks from stem cell infusion up to three months, every 3 months thereafter for the first year post-transplant, and every 6 months thereafter, with no therapy required in case of normal results.
-Both in HBsAg positive or HCV positive allogeneic transplant patients it is crucial to carefully taper the immunosuppressive therapy post-transplant, in order to avoid severe hepatitis reactivation.
-Similarly, if there is a flare up of hepatitis in case or HCV or HBV (ALT ≥ 500 U/L together with documented increased viral load), the addition of immunosuppressive therapy with close monitoring of LFTs, is strongly recommended, to lower the risk of evolution to fulminant hepatic failure REF).

## Hbv Infection

In case of ALT flares during follow-up (ALT ≥ 10–20 fold upper normal value) together with documented increased viral load, either after allogeneic, or autologous transplant, treatment with lamivudine 100 mg in a single daily oral administration for 12 months (alternative in case of no oral uptake: ganciclovir 10 mg/kg/day i.v.) should be considered. Administration of lamivudine results in rapid and profound suppression of HBV replication preventing flares of viraemia and the risk of life-threatening liver disease. Moreover, lamivudine has no major side effects. However, we do recommend a careful use of this drug in transplant setting, as viraemia rapidly reappears after withdrawal of lamivudine together with possible hepatitis recurrence^29^. This could happen if oral uptake becomes impossible for any reason. Additionally, prolonged therapy with this drug is followed by the emergence of HBV-DNA polymerase mutants that may not be free of serious clinical consequences. As the selection of these mutants is time-dependent, the indication for long-term therapy should balance the expected benefit against the risk that the clinical course may, instead, be worsened by the emergence of mutants. The combination of lamivudine and anti-HBs immunoglobulins has also been adopted in other risk situations, such as liver transplants, with encouraging results. The usual schedule of administration for Hepatitis B Ig G is: 5000 U/day i.v. per dose, given every two weeks, starting on day 0 (SC infusion) for three times, and then every month for six months or more if clinically indicated (hepatitis flares).

## Several Settings Should Be Distinguished

### 

#### a) Auto HSCT in HBsAg positive patients:

For these patients, the past-history of liver disease and viral load (eg ; clinical reactivation of hepatitis during the intervals between courses of chemotherapy) is of paramount importance to anticipate on the course after transplant. If hepatitis worsened between CT courses or after CT withdrawal, the patient is likely to have further exacerbation of hepatitis (reactivation) after HSCT.

*If case of normal or slightly abnormal ALT at HSCT, and no reactivation during previous CT:* accurate monitoring of LFT’s especially after HSCT engraftment and from 3 to 6 months afterwards (immune-reconstitution). If there is no post-HSCT liver disease, then no therapy is required.

- If ALT deteriorates during follow-up (ALT ≥ 500 U/L) together with documented increased viral load) Lamivudine (or ganciclovir in case of impossible oral take) should be administered, combined with Hepatitis B IgG.

*In case of abnormal ALT at SC, with previous reactivation during CT:* The association of lamivudine (or ganciclovir) and Hepatitis B IgG is also recommended.

If there is a flare up of hepatitis, an addition of immunosuppressive therapy with corticosteroids, to be tapered very slowly could be considered followed by close monitoring of LFT’s, to reduce the risk of evolution to fulminant hepatic failure. Firm data on the steroid dosage to be used in this clinical setting are not available, although most clinicians use 2 mg/kg/day for the first 2 weeks, followed by careful and slow tapering. Recently, a schedule of 60 mg/day for 4 days and until prothrombin time has stabilized, followed by 10 mg reduction every four days up to 30 mg, and eventually a daily reduction of 2.5–5 mg every 2 weeks, according to transaminases behavior has been published^30^.

#### b) Allo HSCT in HBsAg positive patients:

Lamivudine and Hepatitis B IgG should be administered as for autologous HSCT and according to the same modalities. In addition, post-transplant immunosuppressive therapy should be carefully tapered, because of the risk of acute reactivation in case of abrupt discontinuation.

#### c) Allo HSCT in patients HBsAg negative with HBsAg positive donor:

Although quite exceptional, it may happen that the only HLA-identical sibling donor for a HBsAg negative leukemic patient is HBsAg positive. Choosing between such a donor versus an unrelated HBsAg negative donor should be carefully considered according to the risk of liver complications versus the risk of more GVHD and more transplant-related mortality in unrelated transplants. If the stem cell donor is HBsAg positive, bone marrow stem cells instead of peripheral SCs, is preferred. since the latter theoretically brings a higher viral load and higher risk for acute and chronic GVHD. On the other hand, this risk should be compared with the possible benefit of more graft versus leukemia effect derived from peripheral hematopoietic stem cells, mainly in patients transplanted in leukemia relapse. Different strategies have been proposed in order to reduce the risk of HBV infection in the recipient:

- administration of anti-HBs immuno-globulins, together with HBV vaccination in the recipient: although this strategy has no major complications, the efficacy to prevent HBV transmission has never been clearly demonstrated.
- Donor treatment with lamivudine before donation: although this approach seems quite safe for the recipient, there are still no available published data to support this assumption. Donor health should also be considered: Lamivudine treatment should be initiated only if clinically indicated for the donor.

In case of seroconversion following transplant (from HBV-DNA negative to HBV-DNA positive and/or HBsAg negative to HBsAg positive), lamivudine (or ganciclovir i.v.) are recommended.

If there is clinical hepatitis, immune-suppressive therapy with corticosteroids as described above should be given

## Hcv Infection

### 

#### a) Auto or Allo-HSCT in patients with HCV-RNA in serum (with or without anti-HCV):

Again, careful consideration of the clinical course of liver disease during CT may help for the management of the post-HSCT course. If normal or slightly abnormal ALT at SC, without any post-HSCT liver disease, then no particular measures are required. If ALT increases during follow-up (ALT ≥ 500 U/L together with documented increased viral load) or clinical hepatitis exacerbation, it is recommended to add immunosuppressive therapy, with corticosteroids to be tapered very slowly, with a close monitoring of LFT’s, to prevent evolution to fulminant hepatic failure.

Additionally, to prevent hepatitis A virus co-infection, it is recommended to administer purified hepatitis A inactivated vaccine, prior to SC transplant and at day +28 post-infusion^31^.

#### b) Allo-HSCT in patients HCV-RNA neg with HCV-RNA or anti-HCV positive donor:

This is an exceptional situation, and donor liver disease should be investigated (LFTs and liver biopsy). If interferon therapy is indicated for the donor and it is possible to postpone HSCT without major consequences for the patient, the possibility should be considered to treat the donor with IFN or with IFN plus ribavirin for 1–3 months, in order to reduce amount of virus transferred to the HSCT patient^26^. Therapy should be withdrawn within two weeks before SC harvest. If there is clinical hepatitis related to HCV infection in the recipient after HSCT (seroconversion from HCV-RNA negative to HCV-RNA positive): corticosteroid therapy is indicated, according to treatment schedule criteria already described.

Whatever the source or modalities of HCV infection (de novo, related or not to donor serology, pretransplant HCV positive patient) one should always keep in mind that post-transplant liver disease can be due to multiple concomitant causes. In this respect, besides viral hepatitis (also from viruses other than HCV), hepatic GVHD, drug toxicity, parenteral nutrition, iron overload, fungal infections, and other less common causes, should always be considered. Liver biopsy should be performed, to help in differential diagnosis, before any treatment is given.

In recipients with active chronic hepatitis, with stable engraftment and no evidence of chronic GVHD, treatment with IFN, with or without nucleotide analogues, can be proposed^19^,^28^. There are limited published data on the role of HCV genotypes on post transplant liver disease. It has been reported that genotype has no impact on the risk of liver failure occurring shortly after transplant^32^, while it seems to influence the outcome of chronic hepatitis^28^.

In case of iron overload, phlebotomy or chelating agents should be administered prior to Iinterferon therapy^33^.

## Conclusions:

Although effective prevention of transfusion-acquired HBV and HCV hepatitis should allow to decrease the occurrence of such transplants in the future, there are still some individual situations where the expected long-term benefit of such transplant may exceed the risk of liver disease. Rapid immune reconstitution and viral load are both involved in the mechanism of acute liver failure after HSCT. Such situations should be managed carefully with a special attention during the first months post-transplant, and at any rapid change in the immunosuppressive regimen. Quantitative DNA methods of follow-up are of paramount importance to follow the viral load.

## Figures and Tables

**Table 1:** Prevalence of serum HbsAg + among Stem Cell Transplant candidates

Source*	**Year**	**Total no.patients**	**No. HbsAg+**	**%**
Locasciulli, et al (4)	1990	145	9	6
Reed, et al. (5)	1991	1791	20	1
Locasciulli, et al. (6)	1994	5788	202	3.5
Chen, et al. (7)	1995	88	25	28
Lau. et al. (8)	1997	224	25	11
